# The impact of regional poverty on public health expenditure efficacy across South Africa’s provinces: investigating the influence of historical economic factors on health

**DOI:** 10.3389/fpubh.2024.1442304

**Published:** 2024-11-18

**Authors:** Msawenkosi Dlamini, Josue Mbonigaba

**Affiliations:** Department of Economics, University of KwaZUlu-Natal, Durban, South Africa

**Keywords:** regional, poverty, health, expenditure, South Africa

## Abstract

**Introduction/objectives:**

More than half of South Africa’s population lives in poverty, with significant health disparities across different regions. This study investigates the effects of regional poverty and historical economic factors on the efficacy of public health expenditure to understand how socioeconomic contexts influence overall public health outcomes.

**Methods:**

Our study utilized annual data from 2005 to 2019 for 9 provinces, drawing from the General Household Survey, Health Systems Trust database, and National Treasury’s Intergovernmental Fiscal Review. The primary health outcome was life expectancy at birth, while public health expenditure *per capita* was the main independent variable. We developed the Provincial Index of Multiple Deprivation to assess poverty, incorporating dimensions such as health, education, and living standards. We employed a two-way fixed effects model to examine the complex relationships between regional poverty, public health spending, and health outcomes.

**Results:**

The study found that poverty levels moderate the impact of public health spending on health outcomes, as evidenced by varying results across different provincial regions. Health outcomes in poorer provinces were less influenced by public health spending than wealthier regions. Additionally, the study established that income *per capita*, along with its lagged values and the lagged values of public health expenditure *per capita*, did not significantly affect health outcomes as measured by life expectancy.

**Conclusion/recommendations:**

The impact of health expenditure in South Africa is influenced by regional poverty levels. To maximize the effectiveness of health spending, equitable, region-specific interventions tailored to address the unique health challenges of each area should be implemented.

## 1 Introduction

Poverty is a significant challenge in South Africa that profoundly affects the well-being and quality of life of a large portion of the population. Based on the upper-middle income poverty line of R1,499 per person per month, the World Bank estimates the country’s poverty rate at 62.2% (1). Furthermore, the extent and severity of poverty vary widely across different regions. These disparities reflect the nation’s historical and structural inequalities, particularly affecting rural and underdeveloped areas.

The apartheid system, designed to segregate Black and White populations, was largely responsible for the significant socioeconomic disparities closely tied to regional inequalities in South Africa (2). During apartheid, Black South Africans were forcibly relocated to underdeveloped rural areas known as homelands or Bantustans, while white South Africans resided in well-developed urban centers (3). Consequently, wealthier provinces were predominantly home to white populations, while most black South Africans resided in poorer provinces. Urban areas benefited from extensive infrastructure and resources. However, the demand for labor in cities led to the creation of informal urban settlements for Black workers, as apartheid laws prohibited racial integration in urban residential neighborhoods (4). In rural regions, land governance varied: Some areas were controlled by traditional chiefs, where land was communally held and private ownership was restricted (5); other rural areas followed conventional governance systems, allowing for private ownership of key assets such as land (5). This divergence in governance structures contributed to stark differences in socioeconomic development across rural regions. These regional and socioeconomic disparities, deeply rooted in apartheid policies, have persisted post-apartheid, maintaining an uneven distribution of wealth and resources across different municipalities and provinces in South Africa (6).

Widespread poverty creates significant health challenges, limiting access to essential items such as proper nutrition, sanitation, and healthcare. This deprivation heightens vulnerability to infectious diseases, chronic conditions, and the hazards associated with violence and accidents (7). Poverty also shapes health behaviors, often resulting in low awareness and diminished demand for healthcare and preventive services (8). As a result, poverty exacerbates health problems and hinders the achievement of optimal health outcomes.

In this context, public health expenditure is vital in influencing health outcomes. However, its effectiveness varies across South Africa’s diverse socioeconomic landscape, with regional poverty levels and other factors affecting the demand for and the supply of health services (9). This raises important questions about the equitable distribution and impact of public health resources in these regions.

Bidzha analyzed data from the South African Demographic and Health Survey to examine the impact of public health expenditure on health outcomes at the provincial level between 2002 and 2012 (10). The study focused on four key health indicators: the infant mortality rate, the child and maternal mortality ratios, and life expectancy at birth. The study employed pooled OLS fixed effects (FE) and random effects (RE) models to examine these relationships. The findings revealed a significant relationship between public health expenditure and only two health outcomes: infant mortality rate and life expectancy at birth.

Hlafa et al. employed data from the Health Systems Trust (HST) to investigate the relationship between public health expenditure and health outcomes across South Africa’s nine provinces from 2002 to 2016 (9). The study measured health outcomes using the under-five mortality rate and life expectancy at birth. The authors employed fixed effects (FE) and random (RE) estimation techniques to analyze the data, accounting for time effects and provincial heterogeneity. The results revealed a positive relationship between public health expenditure and health outcomes, although the strengths of these outcomes varied across provinces.

Makuta and O’Hare (11) carried out a comparative analysis of the relationship between public health expenditure and health outcomes in sub-Saharan Africa from 1996 to 2011, utilizing data sources such as the World Bank, United Nations Development Program, and the World Health Organization. Health outcomes were measured through life expectancy at birth and the mortality rate of those under five. The analysis employed two-stage least squares, controlling for income and education. The study also investigated the interaction between public health expenditure and governance quality to assess whether governance moderated the impact of health expenditure on health outcomes. The findings revealed that public health expenditure positively and significantly affected health outcomes, with improved governance further amplifying this effect.

Similarly, Bunyaminu et al. investigated how health expenditure affects life expectancy in a panel of 43 African countries from 2000 to 2018 (12). The study used data from various sources, such as the World Bank, the United Nations Development Program, and the World Health Organization, to measure health expenditure, life expectancy, and government effectiveness. A dynamic panel generalized method of moments(GMM) estimation technique was applied to control for unobserved heterogeneity and endogeneity in the panel model. The researchers found that health expenditure positively impacts life expectancy. The findings revealed that health expenditure positively affected life expectancy, with government effectiveness further enhancing this relationship.

A common limitation of these studies is the lack of a detailed analysis of regional poverty levels and their impact on public health expenditure’s efficacy. They also overlook historical economic factors, such as past income levels and investments, which shape current health outcomes through access to healthcare, education, sanitation, and housing.

Our argument is supported by Francis and Webster’s study, which explored the interplay between historical economic factors and health outcomes in South Africa by analyzing how poverty, inequality, and health are interconnected and influenced by historical elements like colonialism and apartheid (13). These factors have not only exacerbated poverty and inequality but also impacted health, creating a mutually reinforcing cycle that has worsened over time. However, the study’s limitation lies in its methodology: it employed a qualitative and historical approach utilizing broad historical categories for analysis rather than a multivariate analysis to pinpoint the specific impacts of these historical economic factors on health outcomes.

Our study aims to address these gaps by investigating the impact of regional poverty on the relationship between public health expenditure and health outcomes across South Africa’s provinces. We also explore the potential delayed effects of public health spending and income *per capita*, offering a more nuanced understanding of the factors influencing health in South Africa.

## 2 Theoretical foundation

### 2.1 The Grosman model

Our study builds on the Grossman model (14) to examine the complex relationship between public health expenditure, health, and poverty, focusing on South Africa’s provinces. The model is a pivotal contribution to the economic analysis of health and healthcare, as it treats health as both a consumption and an investment good. It posits that individuals demand healthcare to increase their stock of health capital, enhancing their utility and productivity.

The model assumes that health capital depreciates over time and that individuals can invest in health through medical care and other inputs to maintain or improve their health status (15). It also implies that the optimal level of health depends on the individual’s preferences, income, and the prices of health inputs.

### 2.2 Limitations of the Grossman model

However, the Grossman model has some limitations and challenges, especially when applied to a developing country like South Africa. First, the model is based on a micro-level perspective that focuses on individual choices and outcomes, ignoring the macro-level factors that affect the supply and quality of health services, such as public health expenditure, health system performance, and governance. Second, it does not account for the heterogeneity and diversity of the population and regions, such as differences in income, poverty, and health needs across the provinces.

Third, the model does not consider economic factors’ dynamic and lagged effects on health outcomes, such as the impact of past income and public health expenditure on current health status through various intermediary factors. Therefore, a more comprehensive and nuanced theoretical framework is needed to address these limitations and challenges and capture the multifaceted relationship between public health expenditure, health, and poverty in South Africa.

### 2.3 Theoretical framework and hypotheses

Our study aimed to develop a framework by adapting and extending the Grossman model from its original micro-level focus to a broader macro-level approach. This adaptation was tailored to South Africa, incorporating additional variables and mechanisms. We shifted the focus from individual health outcomes to the provincial level, assessing the aggregate and comparative effects of public health expenditure and poverty across South Africa’s nine provinces, as captured by Equation 1:


(1) $$\mathrm{Health}\;\mathrm{outcome}=h\;\left(\begin{array}{l} \mathrm{income},\;\mathrm{public}\;\mathrm{health}\;\mathrm{expenditure},\;\mathrm{poverty},\;\\ \mathrm{public}\;\mathrm{health}\;\mathrm{expenditure}*\mathrm{poverty},\;\\ \mathrm{lagged}\;\mathrm{public}\;\mathrm{health}\;\mathrm{expenditure},\;\\ \mathrm{lagged}\;\mathrm{income} \end{array}\right)$$

Increased provincial income levels could significantly advance human development and economic growth in South Africa. Higher-income levels could lead to increased consumer spending, investment in local businesses, and improved access to education and healthcare (16). Enhanced economic activity and development could foster improved living conditions, create more employment opportunities, and increase public health funding. Collectively, these factors would thus contribute to improved overall health and well-being by providing access to essential health services, reducing health issues, and improving living standards.

Central to our framework is considering public health expenditure as a crucial variable influencing health outcomes alongside income. Public health expenditure reflects the government’s *per capita* investment in health services, which is crucial to improving the availability, accessibility, and quality of healthcare. This investment directly affects the provision of healthcare services. It indirectly influences health outcomes by shaping the overall health system. Such expenditure contributes to developing and maintaining medical facilities, training healthcare professionals, and implementing public health initiatives. Improving these areas leads to more effective medical care, better preventive measures, enhanced health education, and a stronger healthcare infrastructure, all essential to improving the population’s health.

Our study also analyzed regional poverty levels in our adapted model, as these are indicators of various socioeconomic and environmental factors that significantly impact health in different provinces, including nutrition, sanitation, living conditions, and pollution. The multifaceted nature of poverty often leads to a challenging cycle of adverse health outcomes (8). Due to their exposure to adverse socioeconomic and environmental conditions, individuals living in poverty are typically more susceptible to a range of diseases and health complications.

A key aspect of our study is how regional poverty levels might influence the relationship between public health expenditure and health outcomes. This is particularly relevant as impoverished areas will likely have higher healthcare needs than wealthier regions. For example, in poorer regions such as the Eastern Cape, where poverty rates are high, there may be increased demand for public health services like basic healthcare and infectious disease management. Conversely, regions like Gauteng, with higher average income levels, might have different health priorities and expenditure patterns, such as a greater focus on preventive care and chronic disease management. Given these contrasting scenarios, analyzing the interaction between poverty levels and public health expenditure is crucial, as this significantly shapes each province’s health needs and outcomes.

Furthermore, our study integrated an analysis of the potential delayed effects of public health expenditure and income on health outcomes in our model. This is based on the understanding that the impact of these economic factors might not manifest immediately but could be mediated over time through various factors (17). These intermediary factors act as channels through which past economic activities may influence current health outcomes.

**Figure 1:**
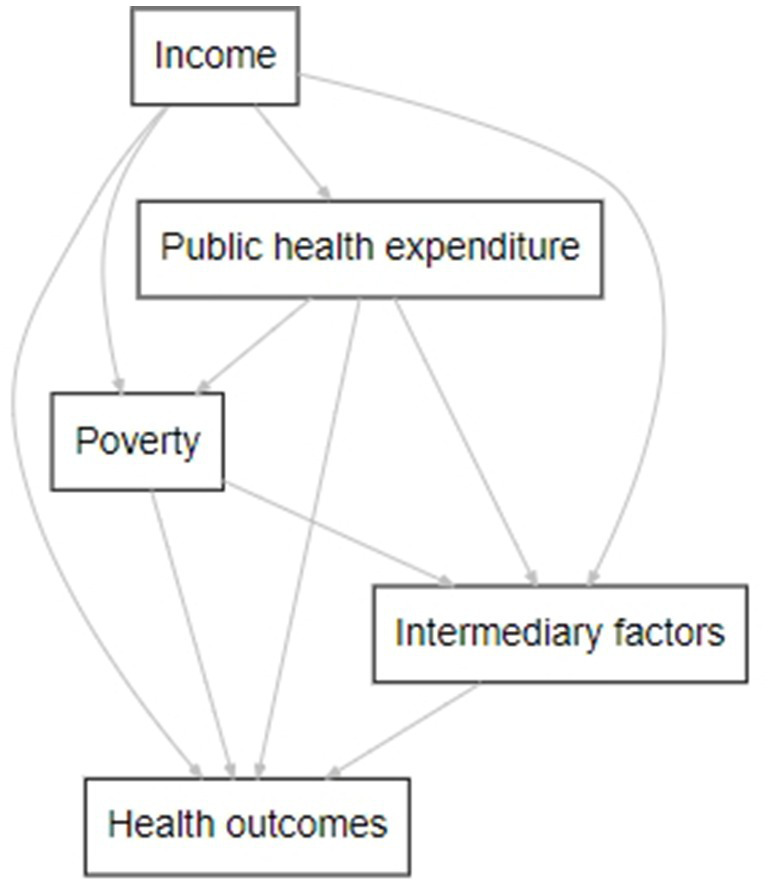
The relationship between public health expenditure, health, and poverty. This figure represents the main variables (nodes) and hypothesized effects (egde) and is the author’s work. It was created using DiagrammeR in R. The direction and strength of these effects will be tested empirically.

Based on our theoretical framework, which is illustrated in Figure 1 below, our study aimed to test the following hypotheses:

*H*1: Poverty levels reduce the effect of public health expenditure on health outcomes.

The hypothesis is formulated based on the observation that regions with higher poverty levels could experience more adverse health outcomes due to poverty compared to wealthier regions, potentially negatively affecting health spending. Consequently, we anticipated variations in this influence across South Africa’s diverse provinces and sought to empirically assess how these differing poverty levels interact with public health expenditure.

*H*2: Historical economic factors, measured by the lag values of income *per capita* and public health expenditure *per capita*, differently impact regional health outcomes.

This hypothesis stemmed from the assumption that economic conditions and health investment in previous years could have lasting effects on the current health status of a population (17).

The following section outlines the empirical approach to test these hypotheses, providing deeper insights into the relationship between public health expenditure, health, and poverty in South Africa.

## 3 Materials and methods

### 3.1 Data

Our study utilized annual data from 2005 to 2019, sourced from various databases, including the General Household Survey (GHS), the Health Systems Trust (HST), and annual reports such as the National Treasury’s Intergovernmental Fiscal Reviews. Table 1 presents the relevant variables and their description. After collecting the necessary information for each province, we merged the data into a single dataset and analyzed the provincial level.

**Table 1:** Description of variables used in the study.

Variable	Description	Categories/values	Used in PIMD
Life expectancy at birth	Represents the average number of years a newborn can expect to live based on current mortality rates.	Life expectancy values for both sexes across all provinces in South Africa.	NO
Provinces	Refers to the different administrative regions within South Africa.	Eastern Cape, Free State, Gauteng, KwaZulu-Natal, Limpopo, Mpumalanga, Northern Cape, North-West, Western Cape.	YES
Public Health Expenditure	Represents total government spending on healthcare services per province.	Continuous	NO
Public Health Expenditure *per capita*	Calculated as total public health expenditure divided by the population of the province.	Continuous	NO
Income *per capita*	Calculated as the GDP in constant prices of each province divided by the size of each province’s population	Continuous	NO
Population Growth rate	Calculated as the percentage difference between the current and previous population.	Continuous	NO
HIV Prevalence	Represents the percentage of the population aged 15–49 estimated to be HIV-positive per province.	Continuous	NO
Female literacy Rate	It represents the percentage of women aged 15 and above who can read and write a short, simple statement with understanding per province.	Continuous	NO
Disability Grant	Indicates whether individuals reported receiving a disability grant.	1: Yes, 2: No	YES
The highest level of education attained	Represents the highest level of education attained by individuals.	Levels of education range from primary (0–8) to high school (8–18), and post-high school qualification (19–24).	YES
Access to Electricity	Indicates whether a household has access to electricity.	1: Yes, 2: No	YES
Fuel for cooking	Represents the type of fuel households use for cooking.	1: Electricity from mains, 2: Electricity from generator, 3: Gas, 4: Paraffin, 5: Wood, 6: Coal, 7: Animal dung, 8: Solar energy.	YES
Access to water	Indicates whether households have access to piped water.	1: Yes, 2: No	YES
Sanitation type	Represents the type of toilet facility used by households.	This ranges from flush toilets (connected to mains or septic tanks) to chemical toilets, pit latrines (with or without ventilation), and bucket toilets. Each can be located in the dwelling, on-site, or offsite.	YES
Housing quality	Represents the type of region in which households reside.	1: Urban formal, 2: Urban informal, 3: Traditional areas, 4: Rural formal.	YES
Asset ownership	A composite variable of several asset variables, including a radio, refrigerator, television, telephone, and car, determines a household’s asset ownership.	1: radio, 2: refrigerator, 3: television, 4: telephone, 5: car.	YES
Unemployment	The official criteria define the unemployment rate per province.	Continuous	YES

Throughout the analysis, our dependent variable was life expectancy at birth, and the primary independent variable under consideration was public health expenditure *per capita*. We opted for public health expenditure *per capita* over total public health expenditure because our dependent variable was life expectancy. As explained in the table above, public health expenditure *per capita* represents the average monetary amount allocated to healthcare for each individual within a population. Consequently, higher *per capita* public health expenditure can lead to improved health outcomes and an increase in the population’s life expectancy. This measure establishes a direct link between the healthcare resources available for each person and their corresponding health outcomes, as indicated by life expectancy.

The next section outlines the methodology used to examine how regional poverty levels affect public health expenditure and influence health outcomes across South Africa’s provinces.

### 3.2 Empirical methods of estimation

This study builds on the methodological approaches of Hlafu et al. (9) by introducing three distinct models, all of which use life expectancy at birth as the dependent variable. The first model focuses on developing the PIMD, drawing inspiration from Noble et al. (18). we employed data from the GHS from 2005 to 2019. The PIMD was designed to capture various dimensions of deprivations faced by individuals, encompassing five critical domains: health, education, standard of living, income, and material deprivation.

Traditional poverty measures, such as income or expenditure, are effective for measuring absolute poverty but fail to capture its multifaceted nature. The PIMD provide a more comprehensive assessment of poverty levels across South Africa’s provinces, transcending conventional money-based metrics. Our primary goal in constructing the PIMD was to capture deprivation consistently over time by selecting indicators from the GHS available from 2005 to 2019.

For example, in the health domain, we used the variable indicating whether an individual receives a disability grant. This measure was chosen because receiving the grant requires a significant health impairment, reflecting the prevalence of health-related deprivation. Households were considered deprived in this domain if at least one member received a disability grant.

In line with the study’s objectives, which mirror the domains in the South African Multidimensional Poverty Index (SAMPI), we incorporated another indicator in the standard of living: the type of fuel used for cooking. This indicator assesses poverty levels by identifying households that rely on basic and potentially hazardous materials such as wood, coal, paraffin, or animal dung for cooking. Dependence on these fuels signals a lower standard of living and limited access to modern amenities, qualifying such a household as deprived in this domain. Table 2 presents a comprehensive overview of all the domains, indicators, and thresholds we employed in constructing the PIMD, ensuring a thorough and nuanced measurement of poverty. The deprivation headcounts for each indicator, calculated using GHS data for each province from 2005 to 2019, are presented in Supplementary material (Tables 17–25).

**Table 2:** Domains, associated indicators, and thresholds of deprivation in the PIMD.

Domain	Indicator	Threshold
Health	Disability Grant	A household is considered deprived in this dimension if at least one member receives a disability grant due to his/her inability to work.
Education	The highest level of education attained	Individuals with less than 5 years’ (Grade 4) formal schooling are considered deprived.
Standard of living	Access to Electricity	Households marked as ‘No’ for having electricity are considered deprived.
	Fuel for cooking	Households using wood, coal, paraffin, or animal dung for cooking are considered deprived.
	Access to water	Households without access to piped water in the dwelling are considered deprived.
	Sanitation type	Households without a flush toilet in the dwelling are considered deprived.
	Housing quality	Households living in informal areas or traditional authority areas are considered deprived.
Income and material deprivation	Asset ownership	A household that does not own more than one radio, refrigerator, television, telephone, or car is considered deprived.
	Unemployment	Households are considered deprived if all adults (aged 15 to 64) are unemployed.

Also integral to this specification is the weighting stage, a crucial aspect of our process that determines the relative importance of each dimension and indicator in assessing the poverty experienced by households in these provinces. In line with the methodology used in the SAMPI and acknowledging that, despite their unique characteristics and challenges, the provinces share fundamentally similar socioeconomic structures, our study implemented a nested weighting system. This entails weighing all domains equally and assigning equal weights to the indicators within each domain.

Table 3 summarizes the details of the weighting structure we employed. The Health and Education indicators are each assigned a weight of 1/4, underscoring their critical role in comprehending the poverty landscape across the provinces. Furthermore, aligned with the SAMPI approach, our study introduced an additional income and material deprivation indicator. This is represented by the Unemployment indicator, which carries a weight of 1/8. Its inclusion is particularly pertinent given its significant impact on income deprivation and material hardship. It thus enhances the index’s robustness and sensitivity, providing a more thorough depiction of poverty’s multifaceted nature.

**Table 3:** Indicators and their weights.

Domain	Indicator	Weight
Health	Disability Grant	1/4
Education	The highest level of education attained	1/4
Standard of Living	Access to Electricity	1/20
	Fuel for cooking	1/20
	Access to water	1/20
	Sanitation type	1/20
	Housing quality	1/20
Income and material deprivation	Asset ownership	1/8
	Unemployment	1/8

Considering that, we multiplied the calculated rates (see Tables 17^25^ in Supplementary material) by their respective weights and combined them to compute a composite score for each province each year. The final step in our specification was to standardize these combined scores, assigning values within a range of 0 to 10. On this scale, zero indicates the least deprived area, and 10 indicates the most deprived area. This facilitates a clear, scaled representation of deprivation levels across the provinces.

In the second specification, we utilized the PIMD figures derived from the previous specification to explore whether regional poverty levels affect the relationship between public health expenditure and overall health across South Africa’s provinces. The study employed the two-way FE model and the subsequent specification to accomplish this. The use of this model is advantageous in this context as it enables control over time-invariant regional characteristics and common time effects. This methodological choice ensures a more nuanced and precise analysis of the intricate interplay between public health spending, regional poverty, and health outcomes.

To implement this approach, our two-way FE model was estimated using Equation 2:


(2) $$\begin{array}{c} LE_{\mathrm{it}}=\beta_{0}+\beta_{1}\ln{\left(\frac{PHE}{\mathrm{pop}}\right)}_{1\mathrm{it}}+\beta_{3}\ln\;{\left(\mathrm{PIMD}\right)}_{3\mathrm{it}}\\ +\beta_{4}\ln\;{\left(GDP\;\mathrm{per}\;\mathrm{capita}\right)}_{4\mathrm{it}}+\beta_{5}\ln\;{\left(PGR\right)}_{5\mathrm{it}}\\ +\beta_{6}\ln{\left(FLR\right)}_{6\mathrm{it}}+\beta_{7}\ln\;{\left(HIV\;\right)}_{7\mathrm{it}}+\beta_{8}\;{\left(GP*\frac{PHE}{\mathrm{pop}}*\mathrm{PIMD}\right)}_{8\mathrm{it}}\\ +\beta_{9}\;{\left(WC*\frac{PHE}{\mathrm{pop}}*\mathrm{PIMD}*\frac{PHE}{\mathrm{pop}}*\mathrm{PIMD}\right)}_{9\mathrm{it}}\\ +\beta_{10}\;{\left(NW*\frac{PHE}{\mathrm{pop}}*\mathrm{PIMD}\right)}_{10\mathrm{it}}+\beta_{11}\;{\left(KZN*\frac{PHE}{\mathrm{pop}}*\mathrm{PIMD}\right)}_{11\mathrm{it}}\\ +\beta_{12}\;{\left(MP*\frac{PHE}{\mathrm{pop}}*\mathrm{PIMD}\right)}_{12\mathrm{it}}+\beta_{13}\;{\left(LP*\frac{PHE}{\mathrm{pop}}*\mathrm{PIMD}\right)}_{13\mathrm{it}}\\ +\beta_{14}\;{\left(FS*\frac{PHE}{\mathrm{pop}}*\mathrm{PIMD}\right)}_{14\mathrm{it}}+\beta_{15}\;{\left(NC*\frac{PHE}{\mathrm{pop}}*\mathrm{PIMD}\right)}_{15\mathrm{it}}\\ +f_{t}+\varepsilon_{\mathrm{it}}\; \end{array}$$

In Equation 2, life expectancy at birth is the dependent variable, measured for each province (i) at time (*t*). The independent variables include the logarithms of the following: public health expenditure *per capita*, GDP *per capita* to estimate income, the population growth rate, the female literacy rate, and the HIV prevalence rate, all specific to each province. Utilizing logarithmic transformations for these variables linearizes their relationships with life expectancy. This creates a more interpretable model that translates effects into percentage changes, accounting for potential skewness in the data distributions.

The PIMD is also included in its original form, specific to each province and period, providing a nuanced view of poverty. Furthermore, Equation 2 included variables to capture time effects ( $f_{t}$ ), which is crucial as it allows the model to account for temporal trends and variations, thereby improving the accuracy and relevance of the findings in a dynamic socioeconomic context (Imai and Kim).

A significant aspect of Equation 2 includes an interaction term between *per capita* public health expenditure, the PIMD, and the provincial dummy variables. This was crucial as it provided insight into how regional poverty levels influenced changes in provincial public health expenditure between 2005 and 2019, compared to the changes experienced by the reference category, namely the Eastern Cape province. It was essential to understand the varying impacts of poverty levels across provinces.

The final specification involved re-estimating Equation 2, but with a key difference: it used lagged values of public health expenditure *per capita* and GDP *per capita* as explanatory variables. This modification was designed to test the possibility that past economic activity could significantly impact the current health status of the population. To conduct this Analysis, we employed an equation similar in structure to Equation 3, adapting it to reflect the influence of these historical economic factors.


(3) $$\begin{array}{c} LE_{\mathrm{it}}=\beta_{1}\ln{\left(GDP\;\mathrm{per}\;\mathrm{capita}\right)}_{i,t-1}+\beta_{2}\ln{\left(GDP\;\mathrm{per}\;\mathrm{capita}\right)}_{i,t-2}\\ +\beta_{3}ln{\left(\frac{PHE}{\mathrm{pop}}\right)}_{i,t-1}+\beta_{4}\ln{\left(\frac{PHE}{\mathrm{pop}}\right)}_{i,t-2}++\beta_{5}\ln\;{\left(GDP\;\mathrm{per}\;\mathrm{capita}\right)}_{5\mathrm{it}}\\ +\beta_{6}\ln\;{\left(PGR\right)}_{6\mathrm{it}}+\beta_{7}{\left(FLR\right)}_{7\mathrm{it}}+\beta_{8}\ln\;{\left(HIV\;\mathrm{prevalence}\right)}_{8\mathrm{it}}\\ +\beta_{9}{\left(\mathrm{PIMD}*\frac{PHE}{\mathrm{pop}}*\mathrm{PIMD}\;\right)}_{9\mathrm{it}}+\beta_{10}\;{\left(GP*\frac{PHE}{\mathrm{pop}}*\mathrm{PIMD}\right)}_{10\mathrm{it}}\\ +\beta_{11}\;{\left(WC*\frac{PHE}{\mathrm{pop}}*\mathrm{PIMD}\right)}_{11\mathrm{it}}+\beta_{12}\;{\left(NW*\frac{PHE}{\mathrm{pop}}*\mathrm{PIMD}\right)}_{12\mathrm{it}}\\ +\beta_{13}\;{\left(KZN\right)}_{13\mathrm{it}}+\beta_{14}\;{\left(MP\right)}_{14\mathrm{it}}+\beta_{15}\;{\left(LP*\frac{PHE}{\mathrm{pop}}*\mathrm{PIMD}\right)}_{15\mathrm{it}}\\ +\beta_{16}\;{\left(FS*\frac{PHE}{\mathrm{pop}}*\mathrm{PIMD}\right)}_{16\mathrm{it}}+\beta_{17}\;{\left(NC*\frac{PHE}{\mathrm{pop}}*\mathrm{PIMD}\right)}_{17\mathrm{it}}\\ +f_{t}+\varepsilon_{\mathrm{it}}\; \end{array}$$

Researchers such as Schultz et al. and Ullah et al. posited that incorporating two lags of the independent variables is sufficient to account for the delayed effects of income and public health expenditure (17, 19). Considering this, we included two lags of each variable in our Analysis, as indicated in Equation 3. This allows us to examine the potential impact of past economic activity on population health, providing a more comprehensive understanding of the temporal dynamics.

Lastly, when interpreting the results from a two-way FE model, it is essential to understand that the process involves more than just determining the direction of the coefficients; their magnitude is equally important as it quantifies the extent and significance of the impact of each variable (20). This provides crucial insights into these variables’ practical and measurable influence on the dependent variable. We used R software as the primary computational tool to facilitate this Analysis.

We present a detailed discussion of the statistical findings considering these methodological considerations.

## 4 Results

### 4.1 Descriptive statistics

This section describes the study’s main variables: public health expenditure, public health expenditure *per capita*, life expectancy, income *per capita*, and poverty. We use different methods to compare these variables across South Africa’s nine provinces and examine their trends over time.

#### 4.1.1 Public health expenditure

**Figure 2:**
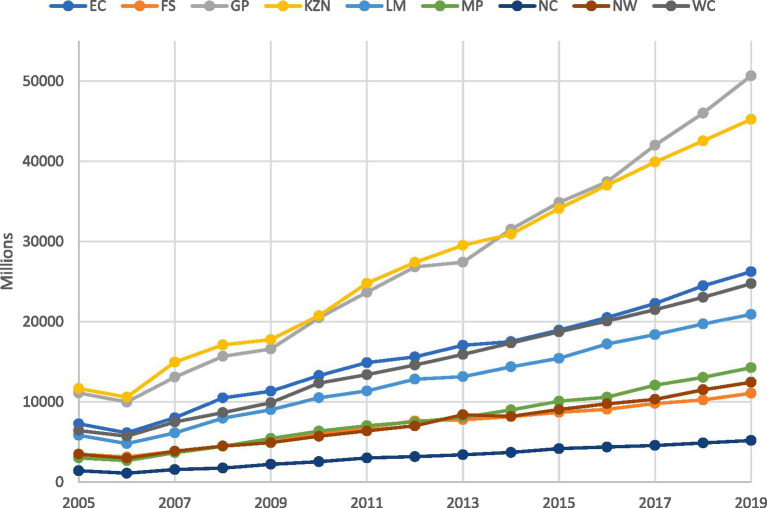
Trends in public health expenditure by province (2005–2019). *This figure is the author’s work and was compiled using the National Treasury’s provincial database data.

There has been a significant increase in South Africa’s public health expenditure over the past decade, primarily focusing on improving access to healthcare among previously disadvantaged populations. This is evident in the data presented in Figure 2, which shows a clear and consistent increase across all provinces and years. Gauteng and KwaZulu-Natal consistently emerge as the top spenders during these 14 years. In 2005, Gauteng allocated R11.12 billion, and KwaZulu-Natal spent R11.66 billion, reflecting substantial financial commitments. By 2019, Gauteng’s expenditure had surged to R50.67 billion, while KwaZulu-Natal’s had risen to R45.23 billion.

Mpumalanga occupies a middle ground in terms of growth. From health expenditure of R3.013 billion in 2005, it experienced steady growth, reaching R14.259 billion in 2019, representing an increase of approximately R11.246 billion over 14 years. While not as substantial as the growth in provinces like Gauteng or KwaZulu-Natal, this signifies a significant commitment to enhancing public healthcare services in Mpumalanga. At the other end of the spectrum, the Northern Cape showed consistently lower levels of public health expenditure. From 2005 to 2019, its spending on healthcare remained relatively stable yet modest compared to other provinces. The budget allocation started at R1.41 billion in 2005 and increased to R5.18 billion in 2019, indicating a modest growth trajectory.

The increase in public health expenditure across South Africa’s provinces indicates an upward trend in healthcare funding. This may have implications for health outcomes, warranting further exploration. In subsequent analysis, we investigate the potential impact of these expenditure patterns on life expectancy across various provinces, examining the data to discern any correlations or trends.

#### 4.1.2 Life expectancy trends across provinces

**Figure 3:**
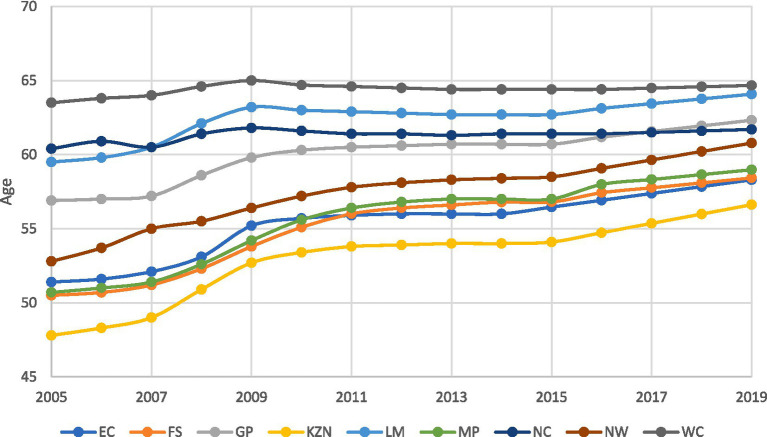
Trends in Life Expectancy by province (2005–2019). *This figure is the author’s work and was compiled using data from the Health Barometers published by the Health Systems Trust.

Building on our understanding of public health expenditure trends, we now shift our focus to the corresponding changes in life expectancy, where, as illustrated in Figure 3, we observe distinct patterns across the provinces.

The Western Cape maintained the highest life expectancy, increasing from 64 to 65 years between 2005 and 2019. While this is a modest increase, it indicates improved healthcare and living conditions in the Western Cape. Notably, this province’s life expectancy aligns with its position among the top provinces regarding public health expenditure (see Figure 2).

Gauteng saw a notable increase in life expectancy, from 57 years in 2005 to 62 years in 2019. This positions it among the top three provinces in terms of life expectancy by around 2018, closely following the Western Cape and Limpopo. The increase in Gauteng’s life expectancy over this period indicates progress in healthcare and overall living conditions within the province.

Despite recording the lowest public health expenditure among all the provinces, the Northern Cape has achieved one of the highest increase in life expectancies, from 53 to 61 years between 2005 and 2019, surpassing five other provinces. In contrast, KwaZulu-Natal, the second-largest spender on public health, recorded the lowest life expectancy. In 2005, life expectancy in this province was 48 years, increasing to 57 years by 2019. This intriguing disparity between provinces’ expenditure and life expectancy outcomes suggests that factors beyond public health expenditure significantly influence life expectancy in these regions.

The life expectancy data for South Africa’s provinces over 14 years reveal notable disparities in health outcomes. While some provinces, such as the Northern Cape, showed significant improvement, others experienced only marginal increases. These variations suggest a complex pattern rather than a uniformly clear upward trend, reflecting diverse levels of improvement in healthcare and living conditions. This nuanced picture underscores the ongoing need for targeted efforts to address regional disparities in access to quality healthcare, ensuring equitable health improvements across all provinces.

Closer examination is required to enhance our understanding of the connection between public health expenditure and life expectancy. The following section presents summary statistics on these variables, offering a deeper perspective on this relationship.

#### 4.1.3 Summary statistics of life expectancy and public health expenditure *per capita*

To deepen our analysis, this section presents a comprehensive summary of statistical data on life expectancy and public health expenditure *per capita* across the provinces. The remainder of the analysis focuses on public health expenditure *per capita*, as it provides a more accurate reflection of individual resource allocation and its impact than aggregate spending.

Table 4 presents the summary statistics of each province’s life expectancy and public health expenditure *per capita*. The table shows life expectancy’s average, minimum, maximum, and standard deviation. The data highlights significant disparities in average life expectancy, ranging from 53 years in KwaZulu-Natal to 64 years in the Western Cape.

**Table 4:** Summary statistics of life expectancy and public health expenditure by province.

	Average life expectancy	Min life expectancy	Max life expectancy	Std dev life expectancy	Average public health expenditure *per capita*	Min public health expenditure *per capita*	Max public health expenditure *per capita*	Std dev public health expenditure *per capita*
EC	55	51	58	2.23	2,888	2054	4,137	680.85
FS	55	51	58	2.78	3,047	1,192	4,790	1156.13
GP	60	57	62	1.76	3,084	1,385	4,910	1122.02
KZN	53	48	57	2.74	2,982	1,591	4,436	922.64
LP	62	60	64	1.38	2,450	1,186	3,667	792.02
MP	56	51	59	2.87	2,291	1,231	3,376	677.84
NC	61	60	62	0.41	3,353	862	5,839	1583.09
NW	57	53	61	2.33	2,338	949	3,659	866.81
WC	64	64	65	0.38	3,423	1,515	5,333	1207.26

*These categorizations are calculated using our data sourced from publications by the National Treasury and the Health Systems Trust.

These disparities underscore the substantial variations in health outcomes among provinces, with standard deviations indicating the degree of variability within each region. While the Western Cape reports the highest average life expectancy, it also displays the lowest standard deviation, suggesting a more consistent life expectancy distribution. Conversely, provinces like KwaZulu-Natal exhibit lower average life expectancy and higher standard deviations, signaling greater variations in health outcomes. These findings are supported by Figure 3 above.

An analysis of public health expenditure *per capita* reveals significant diversity across provinces. Gauteng leads with an average expenditure of R3,084 (see Figure 2), closely followed by the Western Cape at R3,423. In contrast, despite ranking second in aggregate expenditure, KwaZulu-Natal falls fifth in public expenditure *per capita*. This discrepancy can be attributed to the province’s distinct health needs and the fact that it is the second-largest province in South Africa (21).

On the lower end of the scale, the Eastern Cape and Mpumalanga report the lowest average public health expenditure *per capita*, at R2,888 and R2,291, respectively. This contrasts with their rankings based on aggregate expenditure. Similar to KwaZulu-Natal, larger populations and distinct health needs in these provinces could explain the discrepancies observed between Figure 2 and Table 4. Furthermore, the range of public health expenditure *per capita* across provinces is substantial. For example, Gauteng’s maximum *per capita* allocation reached R4,910, while the Eastern Cape’s minimum spending was as low as R2,054 between 2005 and 2019. This wide variation is also reflected in the standard deviation, highlighting disparities in resource allocation among the provinces.

This section explored summary statistics of life expectancy and public health expenditure by province, revealing disparities in resource allocation. The following section examines (YOY) percentage changes to investigate the impact of economic factors on health status and deepen our understanding of these relationships.

#### 4.1.4 Annual trends in health and economic indicators

This section examines annual health and economic indicators trends across various provinces, using year-on-year percentage changes (YoY). These proved a valuable analytical tool, providing insights into the dynamic relationship between historical economic factors such as public health expenditure and income *per capita* and the overall health status within these regions. Our objective was to determine the nature of the relationship between these variables and life expectancy at birth, specifically whether it was non-existent, positive, or negative.

To maintain clarity and focus within the primary results section, we provide the YoY percentage change for provinces with the highest (Gauteng), median (Limpopo), and lowest (Northern Cape) public health expenditure. Detailed data for the other provinces can be found in Supplementary material.

Table 5 presents the annual trends in life expectancy, *per capita* public health expenditure, and *per capita* income in Gauteng, highlighting the YoY percentage changes. The changes reveal the interconnectedness of these factors and their potential impact on health outcomes. Gauteng has consistently improved life expectancy, mainly reflecting positive YoY changes. However, a noteworthy observation in this table is the substantial increase in life expectancy in 2008, marked by a YoY change of 2.45%. This shift suggests that investment in public health expenditure and income in the preceding year may have positively influenced health outcomes, emphasizing the importance of consistent healthcare spending in enhancing life expectancy.

**Table 5:** Annual trends in life expectancy and public health expenditure in Gauteng.

Year	YoY change in life expectancy	YoY change in public health expenditure *per capita*	YoY change in income *per capita*
2005			
2006	0,18	18,19	592,73
2007	0,35	15,39	−42,22
2008	2,45	13,34	−32,32
2009	2,05	11,77	44,01
2010	0,84	4,10	90,26
2011	0,33	15,74	−91,24
2012	0,17	5,24	−8,98
2013	0,17	0,46	4,93
2014	0,00	9,52	−0,17
2015	0,00	16,96	39,62
2016	0,79	6,45	−26,97
2017	0,62	6,06	32,89
2018	0,62	5,71	17,60
2019	0,61	5,41	12,69

*The author’s data was used to calculate YOY percentage changes, following the formula: percentage difference between the current and previous year’s values, divided by the previous year’s value, multiplied by 100.

The data highlights varying trends in public health expenditure *per capita* over time, with a significant rise of 18.19% in 2006. Interestingly, the same year marked the highest annual increase in *per capita* income, showing a remarkable surge of 592.73%. Such variations in these economic factors could influence the accessibility and quality of healthcare services, potentially impacting life expectancy in this province.

However, it is important to note that these economic fluctuations did not immediately reflect in the YOY percentage change in life expectancy for that year or the subsequent one. As noted previously, the effect primarily became evident in 2008. This might suggest a two-year lag in the influence of economic factors working through the intermediaries mentioned earlier, or other factors could have contributed to the surge in life expectancy in 2008.

Moving to Table 6, which shows annual trends in life expectancy, public health expenditure *per capita*, and income *per capita* in the Northern Cape, a notable observation is substantial fluctuations in life expectancy. In 2007, a sharp negative YoY change of −0.66% indicates a decline in life expectancy. However, in 2008, the Northern Cape experienced the highest positive YoY percentage change, with a figure of 1.49%, suggesting that investment in public health or income in that year or the preceding year(s) may have positively influenced health outcomes, akin to the findings for Gauteng.

**Table 6:** Annual trends in life expectancy and public health expenditure in Northern Cape.

Year	YoY change in life expectancy	YoY change in public health expenditure *per capita*	YoY change in income *per capita*
2005			
2006	0,83	41,25	−92,48
2007	−0,66	29,20	518,29
2008	1,49	22,60	−30,67
2009	0,65	18,43	37,31
2010	−0,32	17,77	92,46
2011	−0,32	14,57	−13,86
2012	0,00	10,09	−8,13
2013	−0,16	8,04	−89,59
2014	0,16	7,50	973,15
2015	0,00	12,10	37,52
2016	0,00	8,05	−28,39
2017	0,16	7,43	33,70
2018	0,16	6,91	−88,23
2019	0,16	6,47	11,79

*The author’s data was used to calculate YOY percentage changes, following the formula: percentage difference between the current and previous year’s values, divided by the previous year’s value, multiplied by 100.

Furthermore, the YoY changes in public health expenditure *per capita* reveal considerable volatility, especially in 2007 (29.20%) and 2008 (22.60%), reflecting instability in the allocation of healthcare resources, which can influence healthcare investment for the population in this province.

Regarding *per capita* income in the Northern Cape, 2014 is particularly notable for its extraordinary surge, showing a YOY change of 973.15%. While this indicates improved economic conditions, the data does not readily reveal its immediate impact on life expectancy. Further Analysis may be needed to understand why this specific change in income *per capita* did not translate into improved health in that year or the subsequent year.

Lastly, Table 7 shows the annual trends in life expectancy, public health expenditure *per capita*, and income *per capita* in Limpopo province. Similar to the patterns observed earlier, the most notable increase occurred in 2008, with a substantial YoY change of 2.64%, implying that economic factors in that particular year, along with historical ones, may have played a role in fostering consistent improvements in life expectancy across most of South Africa’s provinces.

**Table 7:** Annual trends in life expectancy and public health expenditure in Limpopo.

Year	YoY change in life expectancy	YoY change in public health expenditure *per capita*	YoY change in income *per capita*
2005			
2006	0,50	14,93	−28,72
2007	1,17	12,99	−39,79
2008	2,64	11,50	−29,32
2009	1,77	10,31	55,83
2010	−0,32	11,56	−80,44
2011	−0,16	11,97	797,21
2012	−0,16	8,11	−6,53
2013	−0,16	2,27	6,86
2014	0,00	8,02	−0,40
2015	0,00	4,63	42,47
2016	0,67	5,99	−24,73
2017	0,51	5,65	36,22
2018	0,50	5,35	21,05
2019	0,50	5,08	13,63

*The author’s data was used to calculate YOY percentage changes, following the formula: percentage difference between the current and previous year’s values, divided by the previous year’s value, multiplied by 100.

As corroborated by Figure 3, public health expenditure *per capita* in Limpopo remains relatively stable compared to the Northern Cape and Gauteng. However, the data also reveals extreme YoY changes in income *per capita*, especially in 2011, where an exceptional positive change of 797.21% was observed.

Interestingly, this substantial increase in income *per capita* does not translate into improved life expectancy, as reflected in the negative −0.16% YoY percentage change in life expectancy for that year and the subsequent one. This disconnection between a significant increase in income and its failure to translate into improved life expectancy highlights the multifaceted nature of this relationship, warranting further examination.

These findings highlight the importance of understanding how extreme income and public health expenditure fluctuations can affect access to healthcare and overall well-being. However, this Analysis does not comprehensively depict the complex relationship between economic factors and individuals’ health in these regions. While there are instances where improvements follow an increase in economic factors in life expectancy, the relationship is not consistently observed. Consequently, the following section delves into the results of a multivariate analysis to gain deeper insight into these complex dynamics.

### 4.2 Results of the provincial index of multiple deprivation

Before examining the results of the multivariate Analysis, it is essential to explore the outcome of the initial specification, which generated the PIMD for each province. This is crucial to understanding the deprivation variations across South Africa’s provinces. Table 8 presents an overview of the PIMD scores by province in South Africa from 2005 to 2019, ranging from 0 to 10. Lower scores indicate less deprivation, while higher scores denote greater deprivation. Analysis of this measure uncovers several significant findings and trends.

**Table 8:** Index of multiple deprivation by province in South Africa (2005–2019).

	2005	2006	2007	2008	2009	2010	2011	2012	2013	2014	2015	2016	2017	2018	2019
EC	3,24	3,16	3,07	3,21	2,73	2,60	2,55	2,46	2,40	2,35	2,34	2,28	2,30	2,29	2,28
FS	2,36	2,23	2,18	2,13	1,56	1,41	1,31	1,37	1,35	1,38	1,44	1,42	1,42	1,37	1,41
GP	1,70	1,71	1,66	1,49	1,21	1,16	1,12	1,15	1,10	1,10	1,20	1,16	1,16	1,16	1,21
KZN	3,42	3,21	3,07	2,57	1,95	1,83	1,91	1,84	1,99	1,97	1,95	1,91	1,90	1,82	1,73
LP	3,29	3,19	3,11	3,19	2,51	2,40	2,30	2,27	2,42	2,36	2,28	2,28	2,28	2,26	2,22
MP	3,00	2,96	2,91	2,81	2,17	1,97	1,89	1,81	1,91	1,97	1,99	1,97	1,92	1,85	1,93
NC	2,19	2,37	2,36	2,23	1,82	1,73	1,84	1,73	1,69	1,68	1,70	1,69	1,69	1,56	1,49
NW	2,75	2,78	2,64	2,66	2,02	1,94	1,85	1,83	1,83	1,86	2,00	1,88	1,83	1,83	1,86
WC	1,60	1,58	1,56	1,36	1,04	0,95	0,90	0,81	0,86	0,84	0,90	0,90	0,89	0,86	0,80

*The content presented here, including calculating deprivation scores using the first specification, is the author’s original work.

First, we observe substantial variation in deprivation levels across provinces. In 2005, the Eastern Cape had the highest level of deprivation, with a score of 3.24, while the Western Cape had the lowest at 1.60. These disparities persisted over the years, with the Eastern Cape consistently having the highest deprivation scores and the Western Cape maintaining its position as the least deprived province.

Second, the data reveals fluctuations in deprivation levels within each province over time. While some provinces like Gauteng show relatively stable scores over the years, others, such as KwaZulu-Natal and Limpopo, experience more variability. KwaZulu-Natal, for example, exhibits a notable decrease in deprivation from 3.42 in 2005 to 1.73 in 2019.

Third, the data shows a consistent decrease in deprivation scores across all provinces, suggesting enhanced living conditions. However, it is crucial to note that the rate of improvement varies. For example, KwaZulu-Natal has the highest annual improvement rate at 0.100, whereas Gauteng has the lowest at 0.034. This indicates that while progress is being made, it is unevenly distributed, highlighting the need for targeted interventions in areas lagging.

The variations in deprivation scores have significant implications for our analysis of how poverty affects the effectiveness of public health expenditure, using the PIMD scores as a measure of poverty. These results are presented in the following section.

### 4.3 Two-way fixed effects regression results: analysis of the base model

The results from the two-way FE model provide insights into the relationship between public health expenditure, regional poverty levels, and life expectancy across South Africa’s provinces.

As anticipated, the coefficient for the logarithm of income *per capita* is positive, signifying that a 1% increase in income *per capita* corresponds to a modest increase of 0.0523 units (years) in life expectancy. However, it is important to note that this coefficient is statistically insignificant, implying no substantial statistical relationship with the dependent variable.

Interestingly, the negative coefficient for the logarithm of public health expenditure *per capita* indicates that a 1% increase in public health expenditure *per capita* leads to an approximate decrease of 2.64 years in life expectancy. This counterintuitive finding suggests that increased public health spending does not directly translate to improved life expectancy. However, this variable is statistically significant at all conventional levels, signifying a significant statistical relationship with the dependent variable. Therefore, further investigation into this relationship is warranted.

The negative coefficient for the PIMD suggests that an increase in poverty levels by one unit corresponds to a decrease in life expectancy by approximately 3.22 units. The substantial magnitude of the PIMD coefficient underscores the significant and direct impact of poverty on life expectancy, indicating that higher poverty levels correlate with considerably reduced life expectancy across South Africa’s provinces. This observation aligns with expectations and reinforces the well-established link between poverty and lower life expectancy. Notably, this variable is statistically significant at all conventional levels, suggesting a robust relationship with the dependent variable (Table 9).

**Table 9:** Two-way fixed effects model results.

Variables	Coefficients	Std. error
The logarithm of Income *per Capita*	0.0523	0.0671
The logarithm of the Population Growth Rate	0.0680	0.0896
The logarithm of the Female Literacy Rate	−0.2172	2.1069
The logarithm of Public Health Expenditure *per Capita*	−2.6415***	0.8303
Logarithm of HIV Prevalence (Ages 15–49)	−1.1772	1.2709
PIMD	−3.2160***	0.7876
Interaction Term: Public Health Expenditure, PIMD, and Free State	0.0010***	0.0002
Interaction Term: Public Health Expenditure, PIMD, and Gauteng	0.0006**	0.0003
Interaction Term: Public Health Expenditure, PIMD, and KwaZulu-Natal	0.0005**	0.0002
Interaction Term: Public Health Expenditure, PIMD, and Limpopo	−0.0003	0.0002
Interaction Term: Public Health Expenditure, PIMD, and Mpumalanga	0.0009***	0.0002
Interaction Term: Public Health Expenditure, PIMD, and North-West	0.0007**	0.0002
Interaction Term: Public Health Expenditure, PIMD, and Northern Cape	−0.0002	0.0002
Interaction Term: Public Health Expenditure, PIMD, and Western Cape	−0.0011***	0.0004

*Equation 2 presents the results presented here. Coefficients marked with * indicate significance at the 10% level, those marked with ** denote significance at the 5% level, and coefficients with *** represent a high significance level at the 1% level.

The analysis of the interaction term between public health expenditure *per capita*, poverty, and the respective provinces yields notable results. Considering regional poverty rates in the Free State, Gauteng, KwaZulu-Natal, Mpumalanga, and North-West, a 1% increase in public health expenditure is linked to increased life expectancy. Specifically, the increases are approximately 0.0010, 0.0006, 0.0005, 0.0009, and 0.0007 years, respectively, compared to the Eastern Cape. These variables were found to be statistically significant, indicating that they have a statistical relationship with life expectancy.

In contrast, the Western Cape was the only province demonstrating a significant negative relationship between *per capita* public health expenditure and life expectancy. Here, a 1% increase in public health expenditure *per capita* is associated with a reduction of 0.0011 years in life expectancy compared to the Eastern Cape. However, it is important to note that the coefficients for these interaction variables are relatively small compared to those for variables like *per capita* public health expenditure and the PIMD. This suggests that the influence of these interaction term variables on life expectancy is comparatively modest.

These results highlight the complex interplay between public health expenditure, poverty levels, and life expectancy across different provinces in South Africa and how these relationships differ from one province to another. The following section examines whether historical economic factors influence life expectancy.

### 4.4 Two-way fixed effects regression results: analysis of past economic activities

The findings presented in Table 10, which include lagged effects of past economic activities, align with those in the base model discussed in the previous section.

**Table 10:** Two-way fixed effects model results.

Variables	Coefficients	Std. error
The logarithm of Income *per Capita*	0.0208	0.0731
The logarithm of the Population Growth Rate	0.1587*	0.0968
The logarithm of the Female Literacy Rate	−2.4690	2.5333
The logarithm of Public Health Expenditure *per Capita*	−2.4519*	1.1736
Logarithm of HIV Prevalence (Ages 15–49)	−3.4625*	1.4236
PIMD	−4.2100***	1.0910
Interaction Term: Public Health Expenditure, PIMD, and Free State	0.0011***	0.0002
Interaction Term: Public Health Expenditure, PIMD, and Gauteng	0.0006*	0.0003
Interaction Term: Public Health Expenditure, PIMD, and KwaZulu-Natal	0.0007***	0.0002
Interaction Term: Public Health Expenditure, PIMD, and Limpopo	−0.0003	0.0002
Interaction Term: Public Health Expenditure, PIMD, and Mpumalanga	0.0009***	0.0003
Interaction Term: Public Health Expenditure, PIMD, and North-West	0.0006*	0.0003
Interaction Term: Public Health Expenditure, PIMD, and Northern Cape	−0.0001	0.0002
Interaction Term: Public Health Expenditure, PIMD, and Western Cape	−0.0003	0.0004
One-year Lag of Public Health Expenditure *per Capita*	−0.0004	0.0005
Two-year Lag of Public Health Expenditure *per Capita*	−0.0004	0.0004
One-year Lag of Income *per Capita*	0.0000	0.0001
Two-year Lag of Income *per Capita*	−0.0001	0.0001

*Equation 3 presents the results presented here. Coefficients marked with * indicate significance at the 10% level, those marked with ** denote significance at the 5% level, and coefficients with *** represent a high significance level at the 1% level.

Regarding the lagged variables of income *per capita*, the coefficient for the one-year lag is 0.0000, implying that in this context, income from a year ago does not significantly impact life expectancy. This variable is also statistically insignificant. In contrast, the two-year lag of income *per capita* suggests that a 1% increase in this variable corresponds to a 0.0001-year reduction in life expectancy. However, the magnitude of this variable is close to zero and insignificant, suggesting that, in this context, income *per capita* from a year and 2 years ago does not significantly impact life expectancy.

Lastly, the one- and two-year lagged public health expenditure *per capita* values show identical magnitudes. This implies that a 1% increase in public health expenditure *per capita* from one and 2 years ago results in a 0.0004-year reduction in life expectancy. However, these values are near zero, and both lagged variables are statistically insignificant. This indicates that, in this context, public health expenditure *per capita* from 1 and 2 years ago does not significantly impact life expectancy.

In conclusion, our findings indicate that while public health expenditure *per capita* significantly affects life expectancy, income *per capita*, its lagged values, and its lagged variables do not influence life expectancy. This conclusion is drawn from the fact that income *per capita* and its lagged values are statistically insignificant, as are the lagged values of public health expenditure *per capita*.

## 5 Discussion

Health spending is widely regarded as a key policy tool, with calls for increased investment as part of the global effort to achieve universal access to healthcare. The rationale is straightforward: more spending leads to expanded health services and infrastructure for the population. These initiatives align with global goals, particularly the Sustainable Development Goals (SDGs). The positive impact of health spending on health outcomes has been well-established. However, evidence suggests that the effects of health spending vary across regions with different development levels (22). In low-development areas, spending often has more immediate and tangible benefits, though inefficiencies and inadequate infrastructure frequently hamper its effectiveness. Since socioeconomic status is a crucial determinant of health, high poverty levels—common in less developed regions—can undermine the potential benefits of health spending. In South Africa, research indicates that these challenges persist, with poverty exacerbating the limitations of public health expenditure in underdeveloped areas.

This study examined the intricate interplay between public health expenditure, regional poverty levels, and health outcomes across South Africa’s provinces. Our findings reveal a nuanced and complex relationship significantly shaped by the varying poverty levels as measured by the PIMD.

We established the the provincial index of multiple deprivation (PIMD) for each of the nine provinces, covering the period from 2005 to 2019. This index revealed significant variations in poverty levels across the provinces. For instance, in 2005, the Eastern Cape had the highest deprivation score at 3.24, in stark contrast with the Western Cape’s score of 1.60. these disparities, initially observed in 2005, persisted throughout the study period. Analyzing the PIMD alongside the Human Development Index (HDI) from the Global Data Lab (1990–2021) (23) and the South African multidimensional poverty index (SAMPI) from Stats SA (based on 2001 and 2011 census data), we identified consistent patterns of poverty variation between the two provinces over time.

After estimating the two-way FE model of the base model, many variables displayed the anticipated signs; however, contrary to expectations, our results indicated that for every 1% increase in public health expenditure *per capita*, life expectancy was projected to decrease by 2.6415 years. This unexpected outcome necessitated further exploration, as it implies that investment in healthcare services per person does not yield the expected life expectancy improvements. This may signal inefficiencies, misallocation of funds, systemic issues, and poor healthcare governance (23), as higher healthcare spending should ideally lead to better health outcomes and longer life expectancy. Indeed, studies found that government effectiveness moderated the impact of public health expenditure on health outcomes in different African contexts (11, 12).

Another possible explanation for these unexpected results is the principle of health persistence discussed by Miller, who posits that regions with lower life expectancy often necessitate increased healthcare spending to address poor health outcomes (24). This scenario is particularly relevant in South Africa, where more than half the population lives in poverty. Longstanding health challenges compromise the efficacy of current public health expenditure in economically disadvantaged areas. Consequently, the observed negative correlation might not imply that higher spending leads to shorter lifespans. Rather, it could indicate that areas with lower life expectancy must invest more in public health, primarily in response to persistent health issues. This finding contrasts with Hlafu et al., who reported a positive relationship between life expectancy and public health expenditure *per capita* in the Western Cape (9). However, it is important to acknowledge that Hlafu et al. did not account for regional poverty levels in their study (9).

A consistent observation emerged in our analysis of historical economic factors, which we measured using one- and two-year lags for public health expenditure and income *per capita*. Both the lag variables for public health expenditure exhibited the same negative coefficient of 0.0004, close to zero. Moreover, these variables were statistically insignificant, implying that historical public health expenditure did not significantly influence life expectancy. Instead, only immediate health expenditure *per capita* appears to impact the dependent variable.

This finding is particularly intriguing when contrasted with research by Ullah et al. that identified a significant relationship between the lagged values of public health expenditure *per capita* and health outcomes (25). However, although significant, their observed effects were relatively small and tended to diminish with increased lags. This difference highlights the complexity and variability of the factors influencing health outcomes over time.

A similar trend emerged with lagged income *per capita*. The one-year lag had no effect, and the two-year lag’s coefficient was close to zero, both statistically insignificant. This contradicts Sharmar’s findings of a positive relationship between income lags and life expectancy, likely due to differing institutional efficiencies in advanced economies (26).

The varying poverty levels across South Africa’s provinces have significant implications for the impact of public health spending on health outcomes. While health spending is crucial for providing and treating adverse health conditions (27), it does not address the underlying socioeconomic factors contributing to poor health outcomes. A recent study reported, for example, that participants with lower educational and income levels had higher healthcare expenditure and used more healthcare compared to participants with the highest educational and income levels, signifying the adverse socioeconomic conditions in propelling the need for healthcare spending (22). Poorer regions benefit more from additional health spending, but overall health outcomes remain relatively low, highlighting the need for more specific, targeted interventions. This type of intervention is in line with the fact that to narrow gaps in heath developing nations would benefit more from increased health spending than the developed world, highlighting that improvement in health expenditure has been part of the solution to address social disparities in health. These interventions should consider the unique health challenges of each region. If implemented alongside socioeconomic initiatives, such targeted health spending would likely be more effective than current allocation practices, achieving better results and delivering greater benefits (22).

In summary, the analysis demonstrates the varied ways poverty influences the effectiveness of public health expenditure in improving people’s health across various provinces, confirming the validity of our first hypothesis. Furthermore, the effects of historical economic factors were insignificant, leading us to reject our second hypothesis that the lagged values of income *per capita* and public health expenditure *per capita* would impact the dependent variable.

## 6 Conclusion and recommendations

This study explored the intricate relationships between regional poverty levels, public health expenditure, and population health outcomes in South Africa, particularly focusing on the role of historical economic factors. Using data from 2005 to 2019 sourced from the GHS, HST database, and National Treasury’s Intergovernmental Fiscal Review (28, 29), we developed the PIMD. This index was then analyzed using a two-way FE model to examine these complex relationships thoroughly.

The study revealed a surprising negative correlation between life expectancy at birth and public health expenditure *per capita*. This contradicts the conventional assumption that higher healthcare spending improves health outcomes, suggesting possible inefficiencies or misallocation of resources within South Africa’s healthcare system. One possible reason for this is health persistency, where regions with historically lower life expectancy demand more public health spending to address longstanding health issues without significant health improvements.

To address these inefficiencies, the study recommends that the South African government review its healthcare spending to optimize resource allocation. This review should focus on improving healthcare distribution and ensuring that funding is effectively targeted, particularly in regions with historically low life expectancy. A health persistency-focused strategy, which directs resources to areas with enduring health challenges, could help reduce health disparities and improve overall life expectancy.

The study also examined the relationship between public health expenditure, regional poverty, and life expectancy across provinces. It found that increased health spending modestly improved life expectancy when adjusted for poverty in provinces like the Free State, Gauteng, and KwaZulu-Natal. However, in the Western Cape, a paradox emerged, with higher health spending linked to a decline in life expectancy, highlighting the limitations of a uniform national health policy across diverse regions. The study calls for region-specific health policies, especially in regions like the Western Cape, where socioeconomic disparities might reduce the effectiveness of increased health spending.

## Data Availability

Data used in this manuscript are included in Supplementary material. All queries relating to data must be directed to the first author of the paper.
