# Supplementary material for: The impact of regional poverty on public health expenditure efficacy across South Africa’s provinces: investigating the influence of historical economic factors on health
Source: Front Public Health. 2024 Nov 18;12:1442304. doi: 10.3389/fpubh.2024.1442304 (PMC11609170; doi:10.3389/fpubh.2024.1442304)
Supplement: Supplementary file 1 [file Data_Sheet_1.docx]

**Appendix**

**Table 11:** Deprivation headcounts on each of the indicators (2005) (%)

|  | **Disability** | **School Years** | **No Electricity** | **Cooking Fuel** | **No Piped Water** | **No Flush Toilet** | **Disadvantaged Areas** | **Deprived Assets** | **Unemployment** |
| --- | --- | --- | --- | --- | --- | --- | --- | --- | --- |
| EC | 3.57 | 41.37 | 31.45 | 55.01 | 35.76 | 59.21 | 50.30 | 48.08 | 28.30 |
| FS | 4.22 | 36.75 | 11.90 | 32.04 | 13.06 | 33.58 | 13.89 | 32.48 | 32.20 |
| GP | 1.41 | 26.45 | 15.03 | 19.13 | 7.62 | 15.20 | 14.23 | 25.70 | 26.00 |
| KZN | 3.21 | 43.54 | 39.62 | 51.28 | 52.36 | 68.87 | 61.43 | 50.37 | 20.50 |
| LP | 1.57 | 44.20 | 16.86 | 63.27 | 31.96 | 77.12 | 75.50 | 44.25 | 21.90 |
| MP | 1.57 | 43.57 | 18.57 | 55.71 | 20.42 | 61.47 | 49.76 | 37.24 | 30.30 |
| NC | 3.88 | 38.66 | 10.68 | 23.64 | 17.36 | 34.41 | 15.28 | 31.51 | 24.90 |
| NW | 3.00 | 41.03 | 14.21 | 38.07 | 26.00 | 55.01 | 44.80 | 34.54 | 26.20 |
| WC | 3.84 | 30.26 | 7.66 | 10.41 | 14.39 | 6.42 | 6.57 | 18.90 | 22.80 |

***Note**: The percentages for these indicators were calculated using the GHS data from 2005.

**Table 12:** Deprivation headcounts on each of the indicators (2006) (%)

|  | **Disability** | **School Years** | **No Electricity** | **Cooking Fuel** | **No Piped Water** | **No Flush Toilet** | **Disadvantaged Areas** | **Deprived Assets** | **Unemployment** |
| --- | --- | --- | --- | --- | --- | --- | --- | --- | --- |
| EC | 3.49 | 40.59 | 30.97 | 50.88 | 35.47 | 56.86 | 49.31 | 46.45 | 28.60 |
| FS | 3.38 | 35.10 | 11.87 | 28.94 | 11.52 | 32.94 | 13.97 | 28.60 | 32.90 |
| GP | 1.41 | 27.17 | 16.83 | 19.94 | 9.44 | 15.77 | 15.11 | 23.18 | 25.40 |
| KZN | 3.23 | 42.01 | 37.22 | 48.30 | 52.51 | 68.43 | 61.88 | 39.13 | 19.80 |
| LP | 1.65 | 43.54 | 15.39 | 57.79 | 35.48 | 76.89 | 75.88 | 39.81 | 20.20 |
| MP | 1.71 | 43.10 | 17.89 | 50.57 | 30.85 | 61.02 | 51.01 | 33.42 | 28.90 |
| NC | 3.56 | 38.55 | 11.99 | 20.95 | 31.62 | 34.92 | 15.38 | 29.09 | 29.90 |
| NW | 2.59 | 40.59 | 13.46 | 35.65 | 43.75 | 56.25 | 46.12 | 31.95 | 26.20 |
| WC | 3.45 | 29.76 | 8.73 | 10.41 | 16.93 | 5.27 | 6.82 | 17.29 | 23.20 |

***Note**: The percentages for these indicators were calculated using the GHS data from 2006.

**Table 13:** Deprivation headcounts on each of the indicators (2007) (%)

|  | **Disability** | **School Years** | **No Electricity** | **Cooking Fuel** | **No Piped Water** | **No Flush Toilet** | **Disadvantaged Areas** | **Deprived Assets** | **Unemployment** |
| --- | --- | --- | --- | --- | --- | --- | --- | --- | --- |
| EC | 3.59 | 39.14 | 29.37 | 47.06 | 29.08 | 57.08 | 53.34 | 44.81 | 28.80 |
| FS | 4.02 | 35.79 | 11.97 | 27.12 | 12.34 | 30.31 | 13.58 | 24.71 | 32.00 |
| GP | 1.84 | 26.66 | 16.31 | 19.02 | 8.15 | 17.69 | 15.44 | 20.66 | 24.80 |
| KZN | 3.73 | 40.94 | 35.77 | 45.80 | 48.74 | 67.35 | 69.74 | 27.90 | 21.30 |
| LP | 1.90 | 42.14 | 12.79 | 58.88 | 27.22 | 76.20 | 83.20 | 35.38 | 22.20 |
| MP | 1.91 | 42.11 | 16.21 | 48.55 | 31.26 | 58.47 | 54.65 | 29.60 | 31.10 |
| NC | 4.32 | 38.56 | 9.95 | 20.21 | 26.96 | 33.47 | 17.11 | 26.67 | 30.00 |
| NW | 3.20 | 39.63 | 13.34 | 32.02 | 26.67 | 53.58 | 51.66 | 29.36 | 25.00 |
| WC | 3.72 | 29.36 | 6.80 | 8.71 | 15.50 | 7.47 | 5.87 | 15.68 | 25.40 |

***Note**: The percentages for these indicators were calculated using the GHS data from 2007.

**Table 14**: Deprivation headcounts on each of the indicators (2008) (%)

|  | **Disability** | **School Years** | **No Electricity** | **Cooking Fuel** | **No Piped Water** | **No Flush Toilet** | **Disadvantaged Areas** | **Deprived Assets** | **Unemployment** |
| --- | --- | --- | --- | --- | --- | --- | --- | --- | --- |
| EC | 3.37 | 40.97 | 31.04 | 46.15 | 39.36 | 59.55 | 58.65 | 43.18 | 30.80 |
| FS | 3.55 | 35.65 | 9.04 | 18.84 | 11.88 | 34.88 | 17.53 | 20.83 | 34.00 |
| GP | 1.62 | 25.47 | 10.75 | 12.96 | 7.96 | 11.46 | 14.35 | 18.13 | 24.30 |
| KZN | 3.38 | 37.47 | 27.69 | 34.33 | 40.07 | 57.79 | 55.31 | 16.67 | 20.90 |
| LP | 2.12 | 43.39 | 16.43 | 60.19 | 41.58 | 84.82 | 86.28 | 30.94 | 17.80 |
| MP | 1.86 | 41.15 | 14.46 | 43.59 | 35.30 | 64.09 | 58.26 | 25.78 | 26.60 |
| NC | 4.35 | 38.24 | 10.26 | 18.94 | 16.17 | 29.20 | 17.84 | 24.24 | 28.00 |
| NW | 3.38 | 39.80 | 12.47 | 29.33 | 30.12 | 57.21 | 53.31 | 26.77 | 26.60 |
| WC | 2.79 | 25.50 | 6.32 | 5.63 | 8.79 | 6.73 | 8.80 | 14.06 | 23.40 |

***Note**: The percentages for these indicators were calculated using the GHS data from 2008.

**Table 15:** Deprivation headcounts on each of the indicators (2009) (%)

|  | **Disability** | **School Years** | **No Electricity** | **Cooking Fuel** | **No Piped Water** | **No Flush Toilet** | **Disadvantaged Areas** | **Deprived Assets** | **Unemployment** |
| --- | --- | --- | --- | --- | --- | --- | --- | --- | --- |
| EC | 3.36 | 20.95 | 29.11 | 41.47 | 54.40 | 60.34 | 59.11 | 41.54 | 30.40 |
| FS | 4.32 | 16.90 | 6.96 | 16.79 | 6.12 | 28.64 | 16.87 | 16.95 | 35.00 |
| GP | 2.03 | 12.60 | 13.20 | 12.33 | 9.77 | 15.91 | 17.46 | 15.61 | 24.60 |
| KZN | 3.59 | 17.96 | 25.82 | 31.98 | 38.92 | 58.21 | 54.25 | 5.43 | 23.70 |
| LP | 2.22 | 18.54 | 13.87 | 58.23 | 51.53 | 84.13 | 84.70 | 26.50 | 15.90 |
| MP | 2.68 | 17.83 | 12.55 | 41.19 | 25.88 | 65.47 | 58.52 | 21.95 | 29.50 |
| NC | 4.63 | 17.50 | 8.38 | 16.30 | 20.36 | 26.68 | 19.16 | 21.82 | 32.30 |
| NW | 3.24 | 17.79 | 11.16 | 24.41 | 32.74 | 54.01 | 51.31 | 24.17 | 26.00 |
| WC | 3.32 | 12.52 | 8.74 | 6.80 | 8.30 | 6.01 | 8.42 | 12.45 | 23.50 |

***Note**: The percentages for these indicators were calculated using the GHS data from 2009.

**Table 16:** Deprivation headcounts on each of the indicators (2010) (%)

|  | **Disability** | **School Years** | **No Electricity** | **Cooking Fuel** | **No Piped Water** | **No Flush Toilet** | **Disadvantaged Areas** | **Deprived Assets** | **Unemployment** |
| --- | --- | --- | --- | --- | --- | --- | --- | --- | --- |
| EC | 2.52 | 19.34 | 26.73 | 42.04 | 53.30 | 57.96 | 57.96 | 39.91 | 29.10 |
| FS | 3.29 | 16.63 | 6.60 | 12.03 | 5.78 | 28.24 | 16.65 | 13.07 | 32.20 |
| GP | 1.33 | 11.27 | 16.99 | 12.68 | 11.21 | 15.91 | 18.89 | 13.09 | 24.60 |
| KZN | 2.78 | 16.75 | 24.63 | 29.18 | 36.94 | 57.19 | 54.08 | 5.90 | 20.80 |
| LP | 1.80 | 17.42 | 10.86 | 55.61 | 51.14 | 85.47 | 86.80 | 22.07 | 15.90 |
| MP | 1.92 | 16.60 | 11.87 | 35.63 | 23.33 | 62.42 | 56.67 | 18.14 | 26.60 |
| NC | 4.32 | 17.65 | 8.78 | 15.92 | 22.74 | 26.87 | 18.81 | 19.40 | 28.70 |
| NW | 2.74 | 17.95 | 11.11 | 22.84 | 29.50 | 52.25 | 51.19 | 21.58 | 25.20 |
| WC | 2.83 | 10.98 | 11.61 | 4.04 | 7.74 | 5.69 | 7.13 | 10.84 | 22.90 |

***Note**: The percentages for these indicators were calculated using the GHS data from 2010.

**Table 17:** Deprivation headcounts on each of the indicators (2011) (%)

|  | **Disability** | **School Years** | **No Electricity** | **Cooking Fuel** | **No Piped Water** | **No Flush Toilet** | **Disadvantaged Areas** | **Deprived Assets** | **Unemployment** |
| --- | --- | --- | --- | --- | --- | --- | --- | --- | --- |
| EC | 2.78 | 20.17 | 23.75 | 35.51 | 53.36 | 56.94 | 57.04 | 38.27 | 29.20 |
| FS | 3.39 | 16.18 | 5.90 | 9.15 | 5.26 | 25.32 | 16.38 | 9.19 | 31.50 |
| GP | 1.20 | 10.62 | 16.87 | 10.66 | 8.62 | 13.74 | 17.63 | 10.56 | 28.60 |
| KZN | 2.50 | 16.78 | 22.50 | 25.77 | 34.12 | 56.04 | 53.70 | 17.13 | 20.50 |
| LP | 1.88 | 16.70 | 7.89 | 52.06 | 45.73 | 84.56 | 85.95 | 17.63 | 18.80 |
| MP | 1.67 | 16.60 | 10.79 | 31.03 | 22.27 | 62.34 | 57.94 | 14.32 | 26.20 |
| NC | 4.08 | 17.55 | 6.67 | 13.33 | 19.72 | 27.58 | 19.09 | 16.98 | 34.80 |
| NW | 2.72 | 17.27 | 8.92 | 17.94 | 28.52 | 52.45 | 50.48 | 18.99 | 25.40 |
| WC | 2.79 | 11.09 | 12.49 | 3.66 | 6.63 | 6.83 | 6.97 | 9.23 | 20.60 |

***Note**: The percentages for these indicators were calculated using the GHS data from 2011.

**Table 18:** Deprivation headcounts on each of the indicators (2012) (%)

|  | **Disability** | **School Years** | **No Electricity** | **Cooking Fuel** | **No Piped Water** | **No Flush Toilet** | **Disadvantaged Areas** | **Deprived Assets** | **Unemployment** |
| --- | --- | --- | --- | --- | --- | --- | --- | --- | --- |
| EC | 3.03 | 19.30 | 16.76 | 32.30 | 52.49 | 56.17 | 56.78 | 36.64 | 29.60 |
| FS | 3.09 | 15.21 | 5.06 | 9.94 | 6.44 | 26.72 | 15.14 | 17.01 | 30.40 |
| GP | 0.95 | 10.99 | 9.70 | 11.13 | 9.37 | 14.08 | 16.20 | 15.79 | 28.40 |
| KZN | 2.79 | 16.39 | 16.75 | 24.59 | 33.70 | 55.49 | 53.85 | 11.51 | 23.60 |
| LP | 1.76 | 17.02 | 6.63 | 52.77 | 48.56 | 83.38 | 85.17 | 13.19 | 20.10 |
| MP | 1.78 | 16.51 | 7.57 | 28.01 | 21.46 | 59.73 | 56.51 | 10.60 | 28.40 |
| NC | 4.09 | 15.38 | 5.66 | 12.39 | 17.78 | 23.84 | 18.11 | 14.55 | 34.10 |
| NW | 2.60 | 16.39 | 8.81 | 18.94 | 28.16 | 51.86 | 51.59 | 16.40 | 28.40 |
| WC | 2.88 | 9.83 | 2.36 | 2.65 | 7.11 | 6.98 | 7.34 | 7.62 | 21.00 |

***Note**: The percentages for these indicators were calculated using the GHS data from 2012.

**Table 19:** Deprivation headcounts on each of the indicators (2013) (%)

|  | **Disability** | **School Years** | **No Electricity** | **Cooking Fuel** | **No Piped Water** | **No Flush Toilet** | **Disadvantaged Areas** | **Deprived Assets** | **Unemployment** |
| --- | --- | --- | --- | --- | --- | --- | --- | --- | --- |
| EC | 3.00 | 19.53 | 16.46 | 25.80 | 52.93 | 55.98 | 56.51 | 34.81 | 29.10 |
| FS | 3.27 | 15.93 | 4.45 | 8.42 | 5.99 | 25.84 | 14.24 | 14.33 | 31.40 |
| GP | 1.07 | 11.65 | 7.49 | 8.21 | 8.68 | 11.73 | 15.44 | 15.09 | 26.80 |
| KZN | 2.79 | 16.95 | 14.68 | 23.45 | 33.95 | 54.56 | 53.88 | 26.77 | 20.40 |
| LP | 1.67 | 17.35 | 5.18 | 48.70 | 48.46 | 82.45 | 85.42 | 28.74 | 18.90 |
| MP | 1.75 | 16.91 | 6.66 | 22.99 | 20.51 | 58.77 | 56.30 | 22.05 | 27.20 |
| NC | 4.40 | 15.73 | 5.11 | 9.11 | 17.89 | 23.66 | 17.96 | 20.84 | 32.70 |
| NW | 2.86 | 15.90 | 7.08 | 15.49 | 29.61 | 51.18 | 51.09 | 22.25 | 25.20 |
| WC | 2.71 | 10.46 | 2.09 | 2.40 | 7.51 | 7.51 | 7.14 | 11.19 | 21.70 |

***Note**: The percentages for these indicators were calculated using the GHS data from 2013.

**Table 20:** Deprivation headcounts on each of the indicators (2014) (%)

|  | **Disability** | **School Years** | **No Electricity** | **Cooking Fuel** | **No Piped Water** | **No Flush Toilet** | **Disadvantaged Areas** | **Deprived Assets** | **Unemployment** |
| --- | --- | --- | --- | --- | --- | --- | --- | --- | --- |
| EC | 2.85 | 19.56 | 12.76 | 22.82 | 54.40 | 56.31 | 56.56 | 33.43 | 28.40 |
| FS | 2.99 | 16.84 | 3.98 | 7.24 | 6.66 | 25.08 | 14.78 | 13.26 | 34.70 |
| GP | 1.11 | 11.55 | 6.07 | 6.70 | 8.15 | 11.61 | 16.05 | 14.43 | 28.60 |
| KZN | 2.75 | 16.72 | 11.97 | 21.58 | 33.55 | 54.09 | 53.80 | 25.07 | 23.90 |
| LP | 1.69 | 16.55 | 4.18 | 45.53 | 45.53 | 82.94 | 86.42 | 27.25 | 19.30 |
| MP | 1.69 | 17.12 | 6.05 | 22.29 | 20.57 | 59.92 | 57.24 | 22.45 | 31.00 |
| NC | 4.08 | 15.70 | 4.86 | 8.60 | 17.19 | 24.21 | 22.60 | 19.29 | 32.00 |
| NW | 2.80 | 16.66 | 6.83 | 13.38 | 30.81 | 51.29 | 51.25 | 22.23 | 26.50 |
| WC | 2.83 | 10.72 | 1.97 | 2.13 | 6.81 | 6.81 | 6.80 | 10.72 | 20.50 |

***Note**: The percentages for these indicators were calculated using the GHS data from 2014.

**Table 21:** Deprivation headcounts on each of the indicators (2015) (%)

|  | **Disability** | **School Years** | **No Electricity** | **Cooking Fuel** | **No Piped Water** | **No Flush Toilet** | **Disadvantaged Areas** | **Deprived Assets** | **Unemployment** |
| --- | --- | --- | --- | --- | --- | --- | --- | --- | --- |
| EC | 2.81 | 19.02 | 12.89 | 22.77 | 54.53 | 58.11 | 49.82 | 36.05 | 28.40 |
| FS | 2.34 | 17.62 | 6.59 | 8.59 | 8.15 | 27.19 | 16.07 | 15.11 | 33.70 |
| GP | 1.26 | 11.87 | 8.74 | 8.70 | 9.26 | 12.55 | 16.76 | 17.77 | 29.30 |
| KZN | 2.80 | 17.38 | 11.23 | 20.05 | 33.23 | 54.07 | 46.32 | 26.61 | 23.30 |
| LP | 1.52 | 16.36 | 4.44 | 38.49 | 46.13 | 75.83 | 77.61 | 29.36 | 20.00 |
| MP | 1.37 | 16.84 | 8.87 | 24.88 | 24.36 | 58.35 | 58.18 | 22.91 | 30.00 |
| NC | 4.99 | 15.99 | 4.84 | 8.20 | 20.08 | 27.55 | 27.44 | 20.93 | 29.20 |
| NW | 2.73 | 16.66 | 7.74 | 13.34 | 33.89 | 55.17 | 52.17 | 26.15 | 30.50 |
| WC | 2.84 | 11.33 | 1.77 | 2.14 | 10.30 | 7.18 | 5.31 | 11.46 | 21.70 |

***Note**: The percentages for these indicators were calculated using the GHS data from 2015.

**Table 22:** Deprivation headcounts on each of the indicators (2016) (%)

|  | **Disability** | **School Years** | **No Electricity** | **Cooking Fuel** | **No Piped Water** | **No Flush Toilet** | **Disadvantaged Areas** | **Deprived Assets** | **Unemployment** |
| --- | --- | --- | --- | --- | --- | --- | --- | --- | --- |
| EC | 2.65 | 19.10 | 10.99 | 20.14 | 53.79 | 57.66 | 53.12 | 32.80 | 28.20 |
| FS | 2.23 | 16.50 | 5.64 | 8.54 | 10.14 | 24.85 | 16.07 | 16.23 | 34.20 |
| GP | 1.42 | 11.97 | 7.14 | 7.85 | 7.88 | 11.93 | 17.46 | 15.93 | 29.10 |
| KZN | 2.27 | 16.72 | 9.14 | 18.72 | 34.25 | 54.29 | 46.32 | 26.04 | 23.50 |
| LP | 1.63 | 16.60 | 2.88 | 38.63 | 50.09 | 75.97 | 77.61 | 26.14 | 21.90 |
| MP | 1.44 | 15.76 | 8.42 | 23.59 | 24.28 | 58.65 | 58.18 | 23.53 | 30.40 |
| NC | 4.80 | 16.10 | 3.91 | 7.49 | 19.33 | 26.82 | 27.44 | 22.26 | 29.60 |
| NW | 2.43 | 16.25 | 6.88 | 11.65 | 32.83 | 52.59 | 52.17 | 22.55 | 28.10 |
| WC | 2.55 | 11.08 | 1.68 | 2.09 | 11.33 | 7.09 | 6.34 | 12.03 | 21.30 |

***Note**: The percentages for these indicators were calculated using the GHS data from 2016.

**Table 23:** Deprivation headcounts on each of the indicators (2017) (%)

|  | **Disability** | **School Years** | **No Electricity** | **Cooking Fuel** | **No Piped Water** | **No Flush Toilet** | **Disadvantaged Areas** | **Deprived Assets** | **Unemployment** |
| --- | --- | --- | --- | --- | --- | --- | --- | --- | --- |
| EC | 2.52 | 18.43 | 8.57 | 19.88 | 53.47 | 57.77 | 56.42 | 31.16 | 32.20 |
| FS | 2.10 | 16.59 | 4.54 | 7.61 | 9.68 | 24.64 | 16.07 | 15.95 | 35.50 |
| GP | 1.22 | 12.19 | 6.80 | 7.52 | 8.45 | 12.08 | 18.17 | 15.98 | 29.00 |
| KZN | 2.18 | 16.93 | 7.07 | 16.51 | 34.24 | 56.05 | 46.32 | 24.24 | 25.80 |
| LP | 1.46 | 16.71 | 3.55 | 36.16 | 47.20 | 75.10 | 77.61 | 28.41 | 21.60 |
| MP | 1.38 | 14.99 | 6.52 | 23.46 | 22.42 | 57.53 | 58.18 | 22.47 | 31.50 |
| NC | 4.98 | 15.84 | 3.80 | 7.11 | 17.26 | 25.73 | 27.44 | 19.87 | 30.70 |
| NW | 2.04 | 15.55 | 6.81 | 11.05 | 33.10 | 52.03 | 52.17 | 22.30 | 26.50 |
| WC | 2.75 | 11.13 | 1.95 | 1.57 | 10.80 | 6.57 | 6.10 | 11.04 | 21.50 |

***Note**: The percentages for these indicators were calculated using the GHS data from 2017.

**Table 24:** Deprivation headcounts on each of the indicators (2018) (%)

|  | **Disability** | **School Years** | **No Electricity** | **Cooking Fuel** | **No Piped Water** | **No Flush Toilet** | **Disadvantaged Areas** | **Deprived Assets** | **Unemployment** |
| --- | --- | --- | --- | --- | --- | --- | --- | --- | --- |
| EC | 2.69 | 18.24 | 6.95 | 18.23 | 51.82 | 56.32 | 59.72 | 29.30 | 35.10 |
| FS | 2.00 | 16.97 | 3.47 | 7.53 | 11.46 | 24.29 | 16.07 | 13.56 | 32.60 |
| GP | 0.97 | 11.64 | 6.20 | 7.95 | 8.11 | 11.37 | 18.88 | 15.45 | 31.10 |
| KZN | 2.14 | 16.13 | 5.51 | 15.67 | 32.04 | 55.08 | 46.32 | 23.07 | 24.10 |
| LP | 1.83 | 17.29 | 2.53 | 35.24 | 49.68 | 75.94 | 77.61 | 26.93 | 19.60 |
| MP | 1.62 | 14.76 | 5.78 | 23.08 | 21.91 | 57.27 | 58.18 | 20.02 | 28.90 |
| NC | 4.37 | 15.57 | 3.57 | 7.01 | 15.35 | 22.81 | 27.44 | 20.87 | 27.10 |
| NW | 1.86 | 15.44 | 5.69 | 11.52 | 34.20 | 51.90 | 52.17 | 21.91 | 27.70 |
| WC | 2.62 | 11.37 | 1.72 | 1.02 | 10.90 | 7.08 | 5.86 | 10.90 | 19.50 |

***Note**: The percentages for these indicators were calculated using the GHS data from 2018.

**Table 25:** Deprivation headcounts on each of the indicators (2019) (%)

|  | **Disability** | **School Years** | **No Electricity** | **Cooking Fuel** | **No Piped Water** | **No Flush Toilet** | **Disadvantaged Areas** | **Deprived Assets** | **Unemployment** |
| --- | --- | --- | --- | --- | --- | --- | --- | --- | --- |
| EC | 2.00 | 18.57 | 5.98 | 14.96 | 51.68 | 57.62 | 63.02 | 27.5 | 36.10 |
| FS | 1.82 | 16.34 | 5.18 | 6.77 | 11.87 | 26.14 | 16.07 | 17.45 | 32.90 |
| GP | 0.55 | 11.81 | 8.72 | 7.98 | 9.66 | 15.35 | 19.59 | 18.99 | 28.90 |
| KZN | 1.11 | 15.70 | 5.40 | 11.89 | 31.09 | 54.39 | 46.32 | 19.23 | 25.60 |
| LP | 1.05 | 17.48 | 2.83 | 34.99 | 50.23 | 76.37 | 77.61 | 27.14 | 16.50 |
| MP | 1.02 | 16.44 | 5.92 | 23.99 | 22.04 | 57.30 | 58.18 | 20.53 | 32.00 |
| NC | 2.53 | 15.11 | 5.42 | 6.23 | 18.11 | 27.91 | 27.44 | 20.18 | 25.00 |
| NW | 1.47 | 15.23 | 8.85 | 11.75 | 34.23 | 49.55 | 52.17 | 24.63 | 28.10 |
| WC | 1.69 | 10.78 | 1.47 | 0.96 | 9.90 | 5.32 | 5.62 | 10.35 | 19.30 |

***Note**: The percentages for these indicators were calculated using the GHS data from 2019.

**Table 26:** Annual Trends in Life Expectancy and Public Health Expenditure in Eastern Cape

| **Year** | **YoY Change in Life Expectancy** | **YoY Change in Public Health Expenditure per Capita** | **YoY Change in Income per Capita** |
| --- | --- | --- | --- |
| 2005 |  |  |  |
| 2006 | 0,39 | 10,13 | -27,92 |
| 2007 | 0,97 | 9,20 | -37,49 |
| 2008 | 1,92 | 8,43 | -36,38 |
| 2009 | 3,95 | -23,29 | 52,97 |
| 2010 | 0,91 | 9,05 | -80,46 |
| 2011 | 0,36 | 13,16 | 777,01 |
| 2012 | 0,18 | 4,85 | -2,30 |
| 2013 | 0,00 | 8,35 | 6,76 |
| 2014 | 0,00 | -0,10 | 1,37 |
| 2015 | 0,82 | 14,80 | 44,73 |
| 2016 | 0,81 | 6,30 | -26,98 |
| 2017 | 0,81 | 5,93 | 37,72 |
| 2018 | 0,80 | 5,60 | 19,63 |
| 2019 | 0,80 | 5,30 | 13,59 |

***Note:** The author's data was used to calculate YOY percentage changes, following the formula: percentage difference between the current and previous year's values, divided by the previous year's value, multiplied by 100.

**Table 27:** Annual Trends in Life Expectancy and Public Health Expenditure in Free State

| **Year** | **YoY Change in Life Expectancy** | **YoY Change in Public Health Expenditure per Capita** | **YoY Change in Income per Capita** |
| --- | --- | --- | --- |
| 2005 |  |  |  |
| 2006 | 0,40 | 21,56 | -92,53 |
| 2007 | 0,99 | 17,74 | -40,39 |
| 2008 | 2,15 | 15,06 | -29,81 |
| 2009 | 2,87 | 13,09 | 44,59 |
| 2010 | 2,42 | 15,54 | 92,26 |
| 2011 | 1,63 | 9,94 | -13,23 |
| 2012 | 0,71 | 20,07 | -90,90 |
| 2013 | 0,35 | 0,65 | 954,11 |
| 2014 | 0,35 | 5,99 | 2,15 |
| 2015 | 0,00 | 4,15 | 43,96 |
| 2016 | 1,11 | 6,83 | -26,77 |
| 2017 | 0,57 | 6,39 | 38,40 |
| 2018 | 0,57 | 6,01 | 17,54 |
| 2019 | 0,57 | 5,67 | 12,51 |

***Note:** The author's data was used to calculate YOY percentage changes, following the formula: percentage difference between the current and previous year's values, divided by the previous year's value, multiplied by 100.

**Table 28:** Annual Trends in Life Expectancy and Public Health Expenditure in KwaZulu-Natal

| **Year** | **YoY Change in Life Expectancy** | **YoY Change in Public Health Expenditure per Capita** | **YoY Change in Income per Capita** |
| --- | --- | --- | --- |
| 2005 |  |  |  |
| 2006 | 1,05 | 12,77 | -29,76 |
| 2007 | 1,45 | 11,32 | -40,16 |
| 2008 | 3,88 | 10,17 | -93,04 |
| 2009 | 3,54 | 9,23 | 1344,74 |
| 2010 | 1,33 | -4,16 | -81,38 |
| 2011 | 0,75 | 15,80 | 799,45 |
| 2012 | 0,19 | 13,27 | -5,96 |
| 2013 | 0,19 | 7,78 | 6,52 |
| 2014 | 0,00 | 2,52 | 1,30 |
| 2015 | 0,19 | 8,51 | 43,00 |
| 2016 | 1,16 | 5,61 | -26,52 |
| 2017 | 1,15 | 5,31 | 34,68 |
| 2018 | 1,14 | 5,04 | 18,80 |
| 2019 | 1,13 | 4,80 | 12,66 |

***Note:** The author's data was used to calculate YOY percentage changes, following the formula: percentage difference between the current and previous year's values, divided by the previous year's value, multiplied by 100.

**Table 29:** Annual Trends in Life Expectancy and Public Health Expenditure in Mpumalanga

| **Year** | **YoY Change in Life Expectancy** | **YoY Change in Public Health Expenditure per Capita** | **YoY Change in Income per Capita** |
| --- | --- | --- | --- |
| 2005 |  |  |  |
| 2006 | 0,59 | 12,38 | -92,81 |
| 2007 | 0,78 | 11,09 | -37,81 |
| 2008 | 2,33 | 9,98 | 600,68 |
| 2009 | 3,04 | 9,07 | 48,61 |
| 2010 | 2,58 | 14,65 | 93,88 |
| 2011 | 1,44 | 7,76 | -9,52 |
| 2012 | 0,71 | -5,45 | -3,59 |
| 2013 | 0,35 | 7,52 | 3,28 |
| 2014 | 0,00 | 6,91 | 0,33 |
| 2015 | 0,00 | 11,64 | 40,83 |
| 2016 | 1,74 | 5,55 | -27,42 |
| 2017 | 0,57 | 5,26 | 34,14 |
| 2018 | 0,57 | 5,00 | 19,02 |
| 2019 | 0,56 | 4,76 | 12,60 |

***Note:** The author's data was used to calculate YOY percentage changes, following the formula: percentage difference between the current and previous year's values, divided by the previous year's value, multiplied by 100.

**Table 30:** Annual Trends in Life Expectancy and Public Health Expenditure in North-West

| **Year** | **YoY Change in Life Expectancy** | **YoY Change in Public Health Expenditure per Capita** | **YoY Change in Income per Capita** |
| --- | --- | --- | --- |
| 2005 |  |  |  |
| 2006 | 1,70 | 20,50 | -32,22 |
| 2007 | 2,42 | 16,92 | -41,59 |
| 2008 | 0,91 | 14,47 | -31,68 |
| 2009 | 1,62 | 12,64 | 44,50 |
| 2010 | 1,42 | 21,46 | 90,59 |
| 2011 | 1,05 | 3,44 | -10,32 |
| 2012 | 0,52 | 6,42 | -12,92 |
| 2013 | 0,34 | 19,91 | 11,68 |
| 2014 | 0,17 | -2,68 | -90,64 |
| 2015 | 0,17 | 7,25 | 1306,74 |
| 2016 | 0,97 | 6,71 | -27,57 |
| 2017 | 0,96 | 6,29 | 31,18 |
| 2018 | 0,96 | 5,91 | 16,26 |
| 2019 | 0,95 | 5,58 | -88,79 |

***Note:** The author's data was used to calculate YOY percentage changes, following the formula: percentage difference between the current and previous year's values, divided by the previous year's value, multiplied by 100.

**Table 31:** Annual Trends in Life Expectancy and Public Health Expenditure in Western Cape

| **Year** | **YoY Change in Life Expectancy** | **YoY Change in Public Health Expenditure per Capita** | **YoY Change in Income per Capita** |
| --- | --- | --- | --- |
| 2005 |  |  |  |
| 2006 | 0,47 | 17,99 | -30,60 |
| 2007 | 0,31 | 15,25 | -40,71 |
| 2008 | 0,94 | 13,23 | -35,22 |
| 2009 | 0,62 | 11,69 | 41,44 |
| 2010 | -0,46 | 20,41 | 87,28 |
| 2011 | -0,15 | 3,28 | -11,37 |
| 2012 | -0,15 | 2,04 | -7,80 |
| 2013 | -0,16 | 7,65 | 5,17 |
| 2014 | 0,00 | 8,20 | -2,01 |
| 2015 | 0,00 | 10,12 | 40,66 |
| 2016 | 0,00 | 6,43 | -27,13 |
| 2017 | 0,14 | 6,04 | 31,25 |
| 2018 | 0,14 | 5,70 | 17,43 |
| 2019 | 0,14 | 5,39 | 11,70 |

***Note:** The author's data was used to calculate YOY percentage changes, following the formula: percentage difference between the current and previous year's values, divided by the previous year's value, multiplied by 100.
